# Editorial: Odontogenic infection as a complication of dental caries: microbiological and molecular aspects

**DOI:** 10.3389/froh.2024.1385026

**Published:** 2024-02-21

**Authors:** Carla P. Lozano, Mariia O. Faustova, Galina A. Loban

**Affiliations:** ^1^ Institute for Research in Dental Sciences, Faculty of Dentistry, University of Chile, Santiago, Chile; ^2^ Department of Microbiology, Virology and Immunology, Poltava State Medical University, Poltava, Ukraine

**Keywords:** dental caries, odontogenic infections, antimicrobials, antiseptics, anti-inflammatories, probiotics

**Editorial on the Research Topic** Odontogenic infection as a complication of dental caries: microbiological and molecular aspects

An important feature of the functioning of the organs and tissues of the oral cavity is that all processes occurring in it are carried out in the conditions of the constant presence of various microorganisms. Moreover, they can cause the development of pathological processes in the body. Odontogenic infections most often occur as a result of the spread of pathogens from the necrotic pulp, periodontal pockets into the soft tissues of the head and neck (1, 2).

The aim of the Research Topic Odontogenic infection as a complication of dental caries: microbiological and molecular aspects, was to disseminate new trends in diagnosis, prevention, research, and possible novel treatments in relation to dental caries, considered the most prevalent chronic non-communicable disease in the world population (3) and its complications, which can lead to odontogenic infections, among others.

In this research topic, all articles highlight the relationship of the oral microbiome with the main dental diseases that occur in it. This effect may be due to various factors, such as the toxic effect of dental biofilm bacteria (Demkovych et al.), adhesive and biofilm-forming properties of bacteria (Faustova et al.). To increase the effectiveness of strategies for the prevention and treatment of caries and odontogenic infections, the authors studied the effect of probiotics (Ma et al.), antioxidant and membrane-protective substances (Lokes et al.).

Four research articles were accepted, three of them are original research articles on the effect of microorganisms (Ma et al.), and antiseptics (Faustova et al.) to prevent the formation of biofilms of *Streptococcus* bacteria directly related to the initiation and development of dental caries or to post-extraction infectious and inflammatory processes in the oral cavity, and on the other hand, natural chemicals for the treatment of odontogenic infections (Lokes et al.). The fourth article is a mini-review of immune system reactions in periodontal tissues in response to dysbiotic subgingival microbiota (Demkovych et al.).

Until now, the use of *in vitro* models of dental caries has led research into this oral disease, which have only partially and very modestly answered clinical and basic science research questions, such as those related to oral microbiology. Promising strains of lactic acid bacteria have been reported in the literature as potential probiotics for the benefit of systemic health, including oral health, with excellent results (4, 5). As an example of the above but inoculating this type of bacteria (as *L. salivarius*) in an animal model, (Ma et al.) described its benefits in inhibiting the growth, metabolism, and biofilm-forming capacity of the most studied acidogenic bacterium, *S. mutans*, preventing the formation of caries lesions. It also provides promising benefits to the intestinal epithelial mucosal barrier and the animal's immune response.

In recent years, the use of antiseptics has been used as an antimicrobial alternative to address post-extraction complications such as infections and inflammation in the oral cavity due to high bacterial resistance to antibiotics (2, 6). Quaternary ammonium-based antiseptics (as decamethoxin), which have a broad antimicrobial action, have been shown to perform better than chlorhexidine (Faustova et al.), considered the gold standard antimicrobial. That is, they decrease the adherence and biofilm-forming capacity of clinical isolates present under the conditions described above compared to clinical isolates from orally healthy subjects.

In addition to state-of-the-art antiseptics, the use of natural substances with antimicrobial and anti-inflammatory properties has also been proposed. This is to improve the effectiveness of treatment of odontogenic infections, specifically odontogenic phlegmon and maxillofacial abscesses. Lokes et al. tested a natural substance (quercitin) as a single application or in combination with anti-inflammatory/antioxidant substances (ethylmethylhydroxypyridine succinate, ES) or with standard protocols. In this regard, the combination of the standard protocol with quercitin and ES successfully reduced the bacterial load at the site of infection and the hospitalization time by 2 days. It would be interesting to test the combination of quercitin with other antioxidants and with antibiotics.

The development of caries is accompanied by a violation of the colonization resistance of the oral cavity, which leads to an increased risk of developing odontogenic infections and inflammatory diseases of periodontal tissues (7, 8). In this research topic, Demkovych et al. noted that plaque bacteria in the area of the bottom of the gingival sulcus penetrate under the epithelium into the stroma of the connective tissue, causing its inflammation. In the development of inflammatory and destructive processes in periodontal tissues, in addition to the imbalance of the body's protective and adaptive mechanisms, the toxic effect of metabolites of periodontopathogenic microorganisms and the release of inflammatory mediators are important (9). The mini-review by Demkovych et al. clearly shows the contribution of about 6 Gram-negative bacterial species (*Porphyromonas gingivalis*, *Aggregatibacter actinomycetemcomitans*, *Prevotella intermedia*, *Tannerella forsythia*, *Eikenella corrodens*, *Fusobacterium nucleatum*) that increase their counts by more than 100 times compared to their counts in dental plaque biofilm. In general, it has been described that these bacteria generate cellular and tissue damage in periodontal tissue by secreting (some of them) proteolytic enzymes that destroy immunoglobulins, collagen or cause gingival epithelial cells to release matrix metalloproteases (MMP-8 and MMP-9), or release anti-inflammatory cytokines. Therefore, the high pathobiont bacterial load (and its exotoxins and/or endotoxins) associated with periodontal diseases alters the responsiveness of the immune system at the oral level.

In conclusion, odontogenic infections as a complicationі of caries pose a significant threat to public health worldwide. Disease severity indicators and the number of patients are increasing. Therefore, improved primary prevention measures and early intervention are needed to alleviate the burden of these infections on the health care system.
